# Psychometric Properties of the Greek Version of the BPDSI-IV: Insights into Borderline Personality Disorder Severity

**DOI:** 10.3390/jcm14113699

**Published:** 2025-05-25

**Authors:** Ioannis Malogiannis, Irini Soultani, Ifigeneia Zikou, Maria-Evangelia Georgitsi, Ioanna Dimitriou, Alexandra Triantafyllou, Antonis Tsionis, Eleni Giannoulis

**Affiliations:** First Psychiatric Department, Medical School, National and Kapodistrian University of Athens, 11528 Athens, Greeceanttsionis@med.uoa.gr (A.T.)

**Keywords:** borderline personality disorder, BPDSI-IV, psychometric properties, reliability, validity

## Abstract

**Background**: Borderline Personality Disorder (BPD) is a growing health concern, characterized by emotional dysregulation, impulsivity, and unstable interpersonal relationships. One of the core features of BPD is self-harm, which has significant implications for clinical management, risk assessment, and treatment planning. Accurate assessment tools are essential in evaluating symptom severity and identifying individuals at high risk of self-injurious behaviors, thereby guiding clinical interventions effectively. This study aimed to assess the psychometric properties, factor structure, and diagnostic utility of the Greek version of the Borderline Personality Disorder Severity Index-IV (BPDSI-IV), providing preliminary evidence for its reliability and validity. **Methods**: A total of 128 individuals with BPD and 32 healthy controls were assessed using the BPDSI-IV together with the Brief Symptom Inventory-53 (BSI-53), the BPD Checklist, the Rosenberg Self-Esteem Scale, the WHOQOL-BREF, and the Defense Style Questionnaire-40 (DSQ-40). BPD diagnoses were confirmed using the Structured Clinical Interview for DSM-5 Personality Disorders (SCID-5-PD). Internal consistency, confirmatory factor analysis (CFA) of previously suggested models, exploratory and confirmatory bifactor modeling, and validity assessments were conducted. **Results**: The BPDSI-IV showed strong internal consistency (α = 0.92, ωt = 0.96), with most subscales demonstrating adequate reliability. Exploratory bifactor analysis using the Schmid–Leiman transformation supported a model with a dominant severity factor (ωh = 0.69), reinforcing the dimensional nature of BPD. CFA supported this bifactorial approach. BPDSI-IV scores significantly discriminated BPD patients from controls (*p* < 0.001). Strong correlations with measures of psychopathology and self-esteem, and correlations with quality of life further supported its validity. **Conclusions**: The Greek BPDSI-IV demonstrated strong reliability and validity indicators. Structured assessment tools, such as the BPDSI-IV, can enhance early intervention and research on the course of borderline personality disorder symptoms.

## 1. Introduction

Borderline Personality Disorder (BPD) is a complex and heterogeneous condition characterized by emotional instability, impulsivity, disturbed interpersonal relationships, and identity disturbances [1]. Historically, BPD has been conceptualized as a disorder that straddles between neurosis and psychosis [1]. More recent research has refined this view and recognizes BPD as a distinct disorder characterized by emotional, cognitive, interpersonal, and impulsive domains [1,2]. This trend has been especially noted in Western and European countries, including Greece [3].

An increasing trend in BPD symptoms and non-suicidal self-injurious behaviors (NSSI) has been reported in the literature, especially in young adults [4,5,6]. Meta-analytic data report a high lifetime prevalence of NSSI in adolescents (17–23.2%) [7,8]. Studies suggest that these percentages have increased by a factor of three to four over the last 20 years [5,6]. As NSSI are considered early indicators of BPD [9], their increasing prevalence raises concerns about the potential increase in BPD diagnoses in future generations. This trend highlights the importance of focusing on early identification and intervention strategies to slow down the progression of symptoms and improve long-term outcomes for those who are at risk. Given the wide range of symptoms of BPD, reliable and valid assessment tools are essential for clinical and research settings.

The Borderline Personality Disorder Severity Index, 4th Edition (BPDSI-IV), is a semi-structured clinical interview designed to measure the frequency and severity of BPD symptoms over a three-month period. This is a change from the previous version, which assessed symptoms over one year [10]. The BPDSI-IV assesses all nine DSM-5 diagnostic criteria for BPD, including fear of abandonment, identity disturbance, affective instability, and dissociative symptoms [2]. Each of these criteria defines a subscale, measured on a 10-point Likert scale, which provides a detailed evaluation of symptom severity. The BPDSI-IV’s shorter assessment window and its ability to assess symptom severity allow for greater sensitivity in symptom fluctuations, treatment outcomes, and longitudinal studies [11,12].

The BPDSI-IV has undergone primary validation in the Netherlands, and although it is becoming increasingly popular in research, there have been few studies examining its reliability and validity in other countries such as Germany, Finland, and Italy [13,14,15]. However, it has been used in clinical trials in several countries [11].

There is an ongoing debate about whether BPD should be considered a dimensional or categorical disorder, which has implications for the development of assessment tools. While traditional methods categorize BPD according to specific criteria, some research suggests that a dimensional approach may better reflect the complexity of the disorder [16,17]. Taxometric analyses support this perspective, highlighting spectrum-like symptom presentation of BPD [17].

Research supports the strong psychometric properties of the BPDSI-IV, with reliability analyses showing high internal consistency (Cronbach’s Alpha above 0.90) and excellent interrater reliability (intraclass correlation coefficients over 0.93) [4,6,7,8,9]. The BPDSI-IV has also been found to have a strong construct and concurrent validity, correlating significantly with other diagnostic instruments, such as the SCID-II and the BPD Symptom Checklist, as well as measures of general psychopathology such as the SCL-90R [18]. These findings underscore its effectiveness in capturing the severity of BPD symptoms across different clinical settings.

Despite its advantages, the reliability of the BPDSI may also depend on clinician expertise. Adequate training is required to ensure consistency in administration and scoring, with interrater reliability studies indicating variability based on examiner experience [18]. To address potential inconsistencies, clinicians should ask additional clarifying questions and make exclusions where necessary, such as distinguishing between dissociative episodes and substance-induced symptoms. This highlights the importance of interrater reliability, as any differences in scoring could affect the validity of the measure and make it difficult to compare results between clinicians.

BPDSI-IV remains underused in many regions due to a lack of validation studies. To expand its use, systematic validation efforts are needed to ensure that it works accurately in diverse populations. The BPDSI-IV is currently used in the multicenter BPDAS Athens study as one of the effectiveness indicators [19].

The primary objective of this study is to evaluate the psychometric properties of the BPDSI-IV in Greek-speaking individuals, including its convergent, concurrent, and discriminant validity, internal consistency, and factor structure. We hypothesize that individuals diagnosed with BPD will score significantly higher on the BPDSI-IV compared to controls. In addition, we expect the BPDSI-IV to show negative correlations with measures such as the Rosenberg Self-Esteem Scale and the WHOQOL-BREF questionnaire, while showing positive associations with the BPD Symptom Checklist and BSI-53. Furthermore, we expect BPDSI-IV scores to correlate negatively with mature and neurotic defenses (as measured by the DSQ-40) and positively with immature defenses.

Our aim is to provide Greek clinicians and researchers with a standardized, empirically supported tool for assessing the severity of BPD. This research will enrich the broader conversation around BPD assessment by shedding light on how well the BPDSI-IV may work in different cultural and linguistic settings. Ultimately, this work may facilitate more accurate diagnosis and monitoring of BPD, improving clinical outcomes and advancing psychiatric research in Greece and beyond.

We hypothesized that BPDSI-IV scores would show stronger correlations with measures theoretically related to BPD (e.g., BPD Checklist) than with less related measures (e.g., WHOQOL-BREF). We further expected strong positive correlations (>0.60) with psychopathology measures and moderate negative correlations (approximately −0.40 to −0.70) with self-esteem and quality of life measures.

## 2. Materials and Methods

The translation and adaptation of the BPDSI-IV scale into Greek was carried out by Malogiannis et al. (2014), using the Dutch version, standardized by Arnoud Arntz and Josephine Giesen-Bloo [19] at the Department of Clinical Psychological Science, Maastricht University. Upon agreement, two bilingual professionals, speakers of Greek (i.e., target) and English (i.e., source), undertook independent forward translations of the BPDSI-IV into the target language. A reconciled version of the instrument was developed, and a professional bilingual translator performed a backward translation of this reconciled version back into the original language. The back-translation and the original one were compared, and any discrepancies between them led to changes to the reconciled translation in the Greek language. An expert committee reviewed this latest version and gave their feedback. At the next stage, the questionnaire was administered to a small group of patients who volunteered to take part in the cognitive debriefing phase to assess clarity and comprehension of the questionnaire items. After this feedback, the final Greek version was produced. The BPDSI was translated within the framework of a randomized clinical trial, examining the effectiveness of predominantly group schema therapy and a combined approach of individual and group schema therapy for borderline personality disorder [20]. To assess the psychometric properties of the Greek version of the BPDSI-IV, we conducted analyses assessing internal consistency, factor structure, and validity in relation to established measures of psychopathology and personality traits.

### 2.1. Instruments

Several questionnaires were administered, including the BPDSI-IV, the BSI-53, the Defense Style Questionnaire (DSQ-40), the WHOQOL-BREF, the Rosenberg Self-Esteem Scale, and the Borderline Personality Disorder Checklist. We selected these instruments because they represent core constructs theoretically associated with BPD severity: symptom distress, self-esteem, defense mechanisms, and quality of life. All participants were also assessed using the SCID-5 PD diagnostic interviews to confirm diagnosis.

- BPD Checklist [21]: A 47-item self-report instrument that assesses subjective distress caused by BPD symptoms over the past month. It has been translated into several languages and has good psychometric properties. In our sample, Cronbach’s Alpha was α = 0.94.
- SCL 90-R [22]: A 90-item self-report inventory designed to assess a broad range of symptoms. The instrument evaluates nine primary symptom dimensions: Somatization, Obsessive-Compulsive, Interpersonal Sensitivity, Depression, Anxiety, Hostility, Phobic Anxiety, Paranoid Ideation, and Psychoticism. Additionally, it provides three global indices to measure overall psychological distress and is widely used for screening, treatment evaluation, and research purposes. In our sample, α = 0.97, and regarding subscales Somatization: α = 0.89, Obsessive-Compulsive: α = 0.85, Interpersonal Sensitivity: α = 0.8, Depression: α = 0.84, Anxiety: α = 0.84, Hostility: α = 0.87, Phobic Anxiety: α = 0.86, Paranoid Ideation: α = 0.88, and Psychoticism: α = 0.8.
- Rosenberg Self-Esteem Scale [23]: A 10-item self-report scale that measures perceived self-esteem. Higher scores indicate greater self-esteem. In our sample, Cronbach’s Alpha was α = 0.88.
- WHOQOL-BREF [24]: Developed by the World Health Organization, this 26-item questionnaire measures four dimensions of quality of life: physical health, psychological health, social relationships, and environment. Higher scores indicate better quality of life. In our sample, α = 0.86, and regarding domains, Physical Health: α = 0.76, Psychological Health: α = 0.80, Social Relationships: α = 0.83, and Environment: α = 0.84.
- Defense Style Questionnaire [25]: This 40-item questionnaire assesses 20 defense mechanisms, categorized as mature, neurotic, or immature. Responses are scored on a Likert scale from 1 to 9, with higher scores indicating greater use of each defense style. In our sample, α = 0,78, and regarding domains, Mature Defenses: α = 0.74, Neurotic Defenses: α = 0.65, and Immature Defenses: α = 0.81.
- BSI-53 [26]: A 53-item scale assessing psychological symptoms across nine dimensions (e.g., depression, anxiety, and paranoia). It is derived from the longer SCL-90-R and measures current symptom intensity and number of reported symptoms. In our sample, α = 0.96.
- SCID-5 PD [27]: A semi-structured interview for the assessment of DSM-5 personality disorders, used both categorically and dimensionally. It includes the Personality Disorder Screening Questionnaire (SCID-5-SPQ), a self-report screening tool for personality disorders.

### 2.2. Participants

A total of 128 individuals diagnosed with BPD and 32 controls from the local community were recruited from the Specific Sector of BPD in Eginition Hospital. Most participants were of middle socioeconomic status and were recruited mainly from the Athens metropolitan area, covering both urban and suburban populations. All participants completed the BPDSI-IV and the Brief Symptom Inventory-53 (BSI-53), and patients also completed the BPD Checklist, the Rosenberg Self-Esteem Scale, the WHOQOL-BREF, and the Defense Style Questionnaire-40 (DSQ-40). The Structured Clinical Interview for DSM-5 Personality Disorders (SCID-5-PD) was used to confirm the clinical BPD diagnoses. Adults who met the inclusion criteria were informed in writing of the aims of the study and provided signed consent to participate. Participants completed a short demographic questionnaire, and the research instruments listed below. These instruments were administered in the Hospital’s Neuropsychology Department under the supervision of specially trained psychologists. All interviewers underwent an initial standardized training session supervised by two senior clinicians, and interrater reliability was assessed both at the beginning and at two additional time points during the data collection to ensure ongoing consistency. Participants were either already undergoing psychological evaluations or were taking part for research purposes only. After receiving instructions, the participants were familiarized with the BPDSI-IV rating scale. The researcher conducted the interviews, always with a second rater present, and each interview lasted about 45 to 60 min. Both the BPD group and the control group, who completed the BPDSI-IV and BSI-53, followed identical procedures. Once the interviews were completed, the psychologist scored the instruments, followed by extensive data entry and statistical analysis. The results were then carefully reviewed to allow the researcher to compare them with the existing literature, leading to the final report of the study.

### 2.3. Reliability Analysis

To assess interrater reliability, the ratings from two independent raters were analyzed using Intraclass Correlation Coefficients (ICCs) and Cohen’s Kappa. The ICCs were calculated for each item of the BPDSI-IV in both BPD patients and controls, using a two-way random effects model with absolute agreement. In cases where the ICC failed due to insufficient variance or negative values, Cohen’s Kappa was used instead. The internal consistency of the BPDSI-IV and its subscales was assessed using Cronbach’s Alpha (α) and McDonald’s Omega (ω). Additional reliability assessments were performed using the Schmid–Leiman transformation.

### 2.4. Confirmatory Factor Analyses, Exploratory Bifactor Analysis, and Model Comparison

We tested a series of alternative models for the structure of the BPDSI-IV items, including a unidimensional model and three-factor, four-factor, and nine-factor models based on the literature [11,16,28,29]. Given the poor fit indices for these models, we proceeded with an exploratory bifactor analysis using the Schmid–Leiman transformation to further investigate the structure of the scale. This approach allowed us to evaluate the presence of a general BPD severity factor and domain-specific factors [30]. To further test this structure, we conducted a confirmatory factor analysis (CFA) of the bifactor model where all items were loaded on both a general BPD factor and its corresponding specific subscales. Model fit indices were compared across all tested models to identify the best-fitting structure, with particular consideration of RMSEA and SRMR, which are robust to sample size limitations in complex models [31].

Assumption checks with a Kaiser–Meyer–Olkin (KMO) measure and Barlett’s test of sphericity were conducted to ensure the validity of the data analysis.

### 2.5. Validity Assessment

Both convergent and discriminant validity were examined. The BPDSI-IV scores were correlated with the BSI-53, WHOQOL-BREF, Rosenberg Self-Esteem Scale, and the BPD Checklist. Criterion validity was tested with independent t-tests and Mann–Whitney U tests to compare BPDSI-IV scores between the BPD participants and the control group. Partial correlations were also calculated to explore the relationships between BPDSI-IV subscales and other clinical measures, controlling demographic variables such as age, gender, education level, and employment status. Additionally, an analysis of covariance (ANCOVA) was conducted to test group differences in BPDSI-IV total scores between patients and controls, while adjusting for age group and years of education as covariates.

Statistical analysis was performed in R Studio (2024.12.1 +563) using R.

## 3. Results

### 3.1. Sample Characteristics

The study included 161 participants, 129 patients with BPD and 32 controls. Among the clinical sample, 83% were outpatients. A total of 64% were receiving psychotherapy, 58% were receiving pharmacological treatment, and 37% had a comorbid psychiatric disorder (most commonly mood and anxiety disorders). The comorbidities included depressive disorder, bipolar disorder (type I or II), anxiety disorders, eating disorders, and substance or alcohol dependence. Comorbidity diagnoses were established through clinical evaluation conducted by a senior psychiatrist. Treatment modalities were categorized into three groups: (a) pharmacotherapy only, (b) psychotherapy only, and (c) combined treatment (pharmacotherapy and psychotherapy). Sample characteristics are presented in Table 1. It was found that a higher percentage of patients fell into the 17–24 age category (*p* = 0.003, Cramér’s V = 0.302), whereas controls were more commonly found in the 41–48 (*p* = 0.042, Cramér’s V = 0.316) and 49–56 (*p* = 0.050, Cramér’s V = 0.315) age ranges. There were also differences in gender identity, with a few patients identified as Non-Binary (2.3%) or Gender-Fluid (0.8%), while none of the controls reported these identities. There was a small but notable difference, with controls having slightly more years of education than patients (14.6 ± 2.1 vs. 14.1 ± 2.1, *p* = 0.045, Cohen’s d = 0.412).

### 3.2. Interrater Reliability

The Intraclass Correlation Coefficient (ICC) was calculated for all items on the BPDSI-IV and each subscale, while Cohen’s Kappa was used for five items that had missing or negative ICCs. We also looked at percentages of agreement to add another level of reliability for these items. For the BPD group, the overall ICC was an impressive 0.973, and regarding subscales, Abandonment: ICC = 0.991, Relationships: ICC = 0.990, Identity: ICC = 0.950, Impulsivity: ICC = 0.992, Suicidality: ICC = 0.997, Affect Dysregulation: ICC = 0.988, Emptiness: ICC = 0.96, Anger: ICC = 0.998, and Dissociation: ICC = 0.997, indicating excellent reliability. The control group showed a similarly strong ICC of 0.946, and regarding subscales, Abandonment: ICC = 0.997, Relationships: ICC = 0.990, Identity: ICC = 0.964, Impulsivity: ICC = 0.991, Suicidality: ICC = 0.999, Affect Dysregulation: ICC = 0.996, Emptiness: ICC = 0.990, Anger: ICC = 0.994, and Dissociation: ICC = 0.992, also indicating high interrater agreement.

### 3.3. Internal Consistency and Factor Analysis

BPDSI-IV showed strong internal consistency, with Cronbach’s Alpha (α) = 0.92 and McDonald’s Omega Total (ωt) = 0.96, confirming its excellent reliability. Reliability analyses of the subscales showed acceptable to high internal consistency (Table 2).

The Kaiser–Meyer–Olkin (KMO) measure of sampling adequacy was 0.7, indicating an acceptable level of sampling adequacy for factor analysis (above 0.6 is considered acceptable, 0.7 is middling [32]). Individual item KMO values were mostly acceptable (>0.50), with only a few items showing marginal adequacy. Bartlett’s test of sphericity was significant (χ^2^(2415) = 5601.11, *p* < 0.001), indicating that correlations between items were sufficiently large for factor analysis to be appropriate.

We tested several alternative models for the latent structure of the BPDSI-IV, including unidimensional, three-factor, four-factor, and nine-factor models derived from the literature [11,16,28,29], all of which showed suboptimal fits. Exploratory bifactor modeling using the Schmid–Leiman transformation revealed a strong general factor (Omega hierarchical, ωh = 0.69), while the subscales still retained meaningful variance. The general factor explained 30% of the common variance, and the omega total was high (ωt = 0.97), indicating that the most reliable variance was captured by both general and group-level dimensions. Subsequent CFA of the bifactor model yielded the best comparative fit (RMSEA = 0.079, SRMR = 0.081, AIC = 40,103.01), although CFI and TLI remained modest (Table 3). These results support a dimensional interpretation of BPD severity, with preserved clinical interpretability of subscales. All item loadings for the bifactor model are presented in Supplementary Table S1.

### 3.4. BPDSI-IV Group Comparisons

BPD patients scored significantly higher than healthy controls (*p* < 0.001) across all subscales (Table 4).

**Figure 1:**
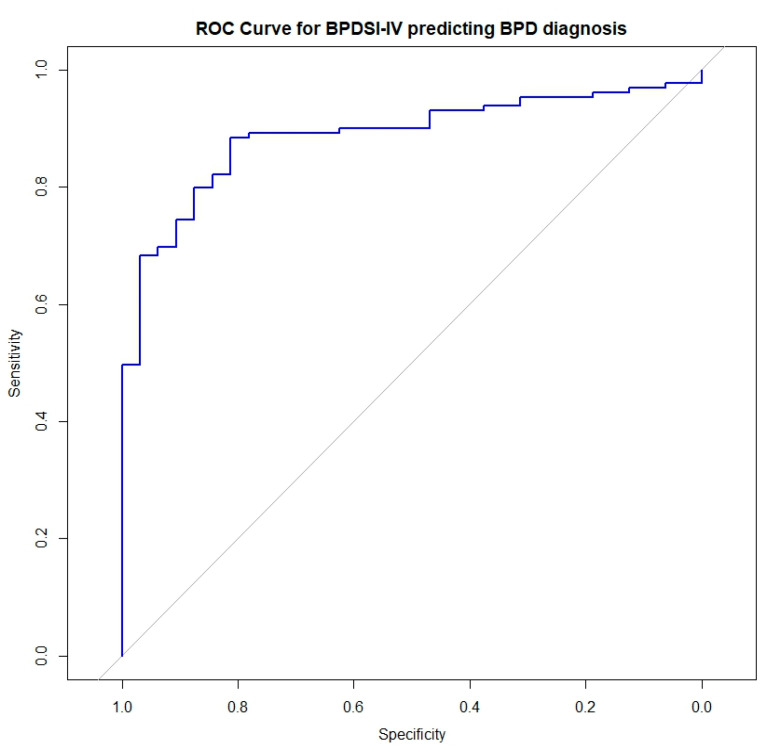
BPDSI-IV: Borderline Personality Disorder Index—IV; BPD: Borderline personality disorder. The blue line represents the Receiver Operating Characteristic (ROC) curve for the BPDSI-IV total score in detecting BPD. The grey diagonal line indicates chance-level performance (AUC = 0.5). The area under the curve (AUC) was 0.90. The optimal cutoff score of 17.88 holds a sensitivity of 90%, specificity of 81.3%, PPV of 95.0%, NPV of 63.4%, and classification accuracy of 87.0%.

The largest effect sizes (Cohen’s d > 1.0) were found for the impulsivity, abandonment fears, and suicidality subscales. ROC curve analysis showed high diagnostic accuracy, with the BPDSI-IV outperforming the BSI-53 in discriminating BPD from controls. The BPDSI-IV yielded an AUC of 0.90, indicating excellent discrimination, whereas the BSI-53 had an AUC of 0.73, reflecting moderate discrimination (Figure 1). The optimal cutoff point, determined by maximizing Youden’s Index, was 17.88, yielding a sensitivity of 90% and a specificity of 81.3%. The positive predictive value was 95.0%, the negative predictive value was 63.4%, and the overall classification accuracy was 87.0%. The moderate NPV was expected, given the enriched prevalence of BPD patients relative to controls in the study sample, which tends to lower the NPV even in well-performing diagnostic instruments. With these results in mind, we propose 17 as a clinical cutoff for BPD in Greek samples.

To assess the severity of BPD symptoms, we categorized BPDSI-IV total scores into three levels: below threshold (<17.88), moderate symptoms (17.88 to 75th percentile), and high burden (>75th percentile). The cutoff of 17.88 was determined through ROC analysis as the optimal point for distinguishing BPD patients from healthy controls. The 75th percentile threshold for high severity was in our dataset 38. In our sample with this classification, 15 patients were presented with low, 82 with moderate, and 32 with severe symptoms. This categorization facilitates a more detailed understanding of symptom severity among BPD patients.

ANCOVA was performed to explore if differences in BPDSI-IV total scores between groups remained significant when adjusting for age group and education years. The differences remained statistically significant (F(1, 157) = 33.92, *p* < 0.001, partial η^2^ = 0.18), indicating a large effect. Age significantly influenced BPDSI-IV scores (F = 8.91, *p* = 0.003, η^2^ = 0.05), and education years had no significant effect (F = 0.42, *p* = 0.516, η^2^ = 0.003). These results confirm that group differences in BPDSI-IV scores are not attributable to demographic variables.

### 3.5. Correlations with Psychopathology and Personality Measures

BPDSI-IV scores showed a strong correlation with the BPD Checklist (r = 0.86, *p* < 0.001), supporting its concurrent validity. Significant correlations were found with SCL-90-R subscales, such as Depression (r = 0.81), Anxiety (r = 0.74), and interpersonal sensitivity (r = 0.79). In terms of personality traits, higher BPDSI-IV scores were associated with a greater use of immature defenses on the DSQ-40 (r = 0.4). Additionally, self-esteem, as measured by the Rosenberg Scale, was negatively correlated (r = −0.69). Finally, individuals with higher BPDSI-IV scores reported a lower quality of life on WHOQOL-BREF (r = −0.4), especially in terms of psychological and social well-being (Table 5).

## 4. Discussion

The aim of this study was to assess the psychometric properties of the Greek version of the Borderline Personality Disorder Severity Index-IV (BPDSI-IV). We examined its internal consistency, factor structure, and overall validity. The BPDSI-IV was found to have excellent internal consistency, strong convergent and criterion validity, and a factor structure consistent with both a multidimensional and a general severity model. Overall, the BPDSI-IV appears to be a reliable and effective instrument for measuring the severity of BPD.

Certain cultural factors (such as collectivistic tendencies and family-centered values prevalent in Greek culture) may influence the expression of abandonment fears and interpersonal sensitivity. Also, during translation, minor adaptations were necessary for items addressing identity and dissociation to preserve conceptual equivalence.

Known-group validity was strongly supported by the BPDSI-IV, which discriminated well between BPD patients and controls, with large effect sizes across most subscales. Cohen’s d for total score differences was 1.40 (*p* < 0.001), and subscale differences ranged from moderate to very large (d = 0.60–1.67), especially for the impulsivity, abandonment, and suicidality domains. Analysis of covariance showed a significant difference in BPDSI-IV total scores between BPD patients and controls, even after adjusting for age and education. The partial eta squared (η^2^ = 0.18) indicated a large effect size, confirming that the BPDSI-IV meaningfully differentiates clinical from non-clinical populations.

Regarding convergent validity, the BPDSI-IV total score showed strong positive correlations with BPD symptom-related instruments such as the BPD Checklist (r = 0.86) and SCL-90-R subscales (r = 0.74–0.81), indicating large effect sizes. Correlations with psychological distress (BSI), self-esteem (Rosenberg Scale), and immature defense mechanisms (DSQ-40) ranged from moderate to strong (r = ±0.4 to ±0.7). The correlations with more adaptive traits, such as mature defenses, were negative and of small-to-moderate magnitude (r = −0.45), supporting the expected discriminant validity pattern. These correlation patterns remained significant after controlling age, gender, and education.

ROC curve analysis confirmed diagnostic accuracy (AUC = 0.90), with high sensitivity (0.88) and specificity (0.81) at the optimal cutoff of 17.88. The positive predictive value (PPV = 95.0%) was high due to the enriched BPD sample, while the negative predictive value (NPV = 63.4%) was moderate, as expected due to the smaller control sample. This suggests that the BPDSI-IV is an effective screening tool, particularly in clinical settings where BPD prevalence is high. While some psychometric properties observed in our study, such as internal consistency, align with those reported by Giesen-Bloo et al. (2010) [11], the dimensionality results differ substantially. Unlike the original study, which favored a nine-factor structure, our findings support a bifactor model characterized by a dominant general severity factor alongside a specific subdomain. These differences may reflect variations in sample characteristics, cultural influences, or methodological approaches, underscoring the need for further cross-cultural validation studies using confirmatory techniques. More specifically, in our study, the BPDSI-IV showed high internal consistency; Giesen-Bloo et al. found an internal consistency of α = 0.96 [11]. The reliability of its subscales varies, with coefficients ranging from moderate to high. However, in our study, the Abandonment-fear (α =0.61) and Dissociation (α = 0.58) subscales showed lower reliability than the original study (α = 0.79 and α = 0.80). Similar figures, particularly for the Dissociation subscale, were reported in the Italian validation study [15]. The lower reliability of the Abandonment and Dissociation subscales may partially stem from cultural norms in Greece, where fear of abandonment is often normalized within tight-knit families, possibly reducing item variance. Additionally, dissociative experiences might be underreported due to the stigma associated with mental health symptoms in Greek society. Furthermore, while the original study tended towards a nine-factor structure, our results support a bifactor model. Although the nine-factor model was previously considered the best fit, it did not perform as well in our study. Instead, the bifactor model provided a better fit, suggesting a general severity factor alongside specific symptom domains. This view is consistent with the modern view of BPD as a spectrum of distress, while still recognizing it as a distinct condition. The subtle differences between this and the original study may be due to differences in sample characteristics or cultural influences on symptom expression.

Borderline personality disorder is increasingly recognized as a significant public health problem due to its high prevalence, chronic nature, and its effects on mental health services [1]. The availability of reliable and valid instruments, such as the BPDSI-IV, is essential for assessing the severity and course of this disorder. This will enable structured clinical approaches and enhance research into effective treatment strategies. There is an urgent need for standardized assessment instruments to improve diagnostic accuracy and support targeted interventions, ultimately improving patient outcomes and lessening the strain on healthcare systems.

The importance of early identification and intervention is emphasized by recent research suggesting that borderline symptoms can emerge in adolescence and young adults (3–7). It has also been reported that BPD symptoms, even subclinical ones, are associated with other psychiatric disorders and functional disability [29]. This finding has further highlighted the need to identify BPD symptoms in both clinical practice and research. The ability of BPDSI-IV to quantify symptom severity, rather than simply categorizing diagnoses, makes it a valuable instrument for clinical decision making and for research into the developmental trajectories of BPD. Early identification of borderline features could help prevent long-term functional problems and psychological distress, emphasizing the need to include BPDSI-IV in regular assessments.

Personality traits may also influence the course and trajectory of other clinical constructs. For example, Rodriguez et al. reported borderline personality traits in patients with persistent depressive disorders [30]. BPD has also shown comorbidity with bipolar disorder, which may sometimes go unnoticed [31]. Considering such reports, the validation of the BPDSI-IV for the Greek public may provide a tool for further investigation and research in other clinical populations. We caution that validation is an ongoing process requiring replication across different populations and contexts; validity is not an inherent property of an instrument but depends on its use and sample characteristics.

Our findings contribute to the growing body of evidence supporting the use of the BPDSI-IV in Greek-speaking populations and open avenues for further investigation in diverse clinical samples. However, we acknowledge that the process of accumulating evidence for the reliability and validity of any psychometric tool is inherently ongoing and context-dependent, requiring continual reassessment across different populations and settings. Some limitations of our study should be noted. First, reliance on a clinical sample with a relatively small control group might restrict the generalizability of our results. Future studies should aim to include larger and more diverse control samples. Second, although the bifactor model demonstrated better fit in contrast to other models, some of the fit indices, particularly the CFI and TLI, did not reach conventional thresholds for good fit. This may be partially attributed to the complexity of the model and the moderate sample size, which can influence the stability of these indices. While RMSEA and SRMR supported acceptable model adequacy, the modest CFI/TLI values suggest that the factor structure of the BPDSI-IV may benefit from further refinement. To address this, we recommend that future research conduct a full EFA in larger and independent samples that could uncover alternative or more culturally specific structures that may improve the psychometric properties of the scale and potentially inform revisions to its item composition or subscale grouping. Lastly, despite its strong psychometric properties, some subscales, particularly those related to fear of abandonment and dissociation, showed relatively lower reliability. Future research should investigate ways to refine these subscales to increase their precision or to explain the results in terms of cultural understanding. With the increasing focus on dimensional models of the personality spectrum, the relevance of the BPDSI-IV within transdiagnostic frameworks needs to be further explored. Third, it is important to note that several key psychometric properties of the BPDSI-IV were not assessed in the present study. Specifically, test–retest reliability was not assessed in the current study, but a follow-up project is underway to examine the test–retest stability and longitudinal sensitivity of the BPDSI-IV in a clinical cohort over a six-month period. Moreover, we did not examine the instrument’s sensitivity to change over time or its predictive validity. These characteristics are critical for determining whether the BPDSI-IV can effectively track symptom progression and forecast clinical outcomes. As such, future studies should focus on longitudinal designs and treatment-monitoring contexts to evaluate the BPDSI-IV’s responsiveness and its potential to inform prognosis and therapeutic decision-making.

In conclusion, the Greek BPDSI-IV demonstrated preliminary evidence of strong reliability and validity. Further studies are needed to expand psychometric support across diverse clinical settings. The bifactor model findings reinforce the presence of a general severity factor while retaining clinically meaningful subscales. These findings highlight the value of the BPDSI-IV as an important tool for both clinical assessment and research regarding BPD and BPD symptoms. In summary, the Greek version of the BPDSI-IV demonstrates strong psychometric properties and appears to be a reliable and contextually appropriate tool for assessing BPD severity. It can serve as a structured assessment tool in specialized psychiatric clinics, help screen for symptom severity in primary care when BPD is suspected, and provide outcome measures in psychotherapy and pharmacological intervention studies within Greek healthcare settings. Further research is warranted to expand the evidence base across diverse populations and clinical settings.

## Figures and Tables

**Table 1:** Sample characteristics and comparisons.

Variables	Patients	Controls	*p*-Value	Effect Size (Cramer’s V/Cohen’s d)
Female (%)	107 (82.9%)	25 (78.1%)	0.528	0.302
Male (%)	18 (14%)	7(21.9%)		
Age group 17–24	63 (48.8%)	11 (34.4%)	0.003 *	0.302
Age group 25–32	28 (21.7%)	4 (12.5%)	0.074	0.211
Age group 33–40	26 (20.2%)	5 (15.6%)	0.070	0.227
Age group 41–48	9 (7.0%)	6 (18.8%)	0.042 *	0.346
Age group 49–56	3 (2.3%)	6	0.050	0.315
Non-Binary (%)	3 (2.3%)	0	-	-
Gender-Fluid (%)	1 (0.8%)	0	-	-
Unemployed (%)	44 (34%)	8 (25%)	0.221	0.080
Students, school (%)	35 (27%)	9 (28%)	0.081	0.185
Pension (%)	1 (0.7%)	0 (0%)	-	-
Education years(Mean ± SD)	14.1 ± 2.1	14.6 ± 2.1	0.045 *	0.412 (Cohen’s d)

* Statistical significance, *p* < 0.05.

**Table 2:** Internal consistency and interrater reliability of the BPDSI-IV scale and its subscales.

Subscale	Cronbach’s Alpha (α)	McDonald’s Omega (ω)	Interclass Correlation Coefficient (Patients/Controls)
BPDSI-IV (Total)	0.92	0.96	0.973/0.946
Abandonment	0.61	0.79	0.991/0.997
Relationships	0.73	0.74	0.990/0.990
Identity distortion	0.77	0.77	0.950/0.964
Impulsivity	0.61	0.75	0.992/0.991
Para(suicide)	0.89	0.9	0.997/0.999
Affective Instability	0.81	0.82	0.988/0.996
Emptiness	0.71	0.74	0.965/0.990
Anger-control	0.81	0.83	0.998/0.994
Dissociation	0.58	0.76	0.997/0.992

**Note:** BPDSI-IV (Total): The total scale of Borderline Personality Disorder Severity Index—forth version.

**Table 3:** Results of the confirmatory factor analysis of different models.

Model	χ^2^ (df)	CFI	TLI	RMSEA	SRMR	AIC	BIC
One-Factor	6936.56 (2345)	0.259	0.237	0.123	0.119	45,357.67	45,958.20
Three-Factor ^a^	5965.52 (2342)	0.415	0.397	0.110	0.141	44,392.63	45,001.77
Four-Factor ^b^	5702.30 (2339)	0.457	0.440	0.106	0.123	44,135.41	44,753.13
Nine-Factor ^c^	5048.42 (2309)	0.558	0.538	0.096	0.088	43,541.53	44,245.04
Bifactor	3978.99 (2239)	0.595	0.563	0.079	0.081	40,103.01	40,800.74

**Note:** Model fit indices for exploratory factor models of the BPDSI-IV. ^a^ Sanislow et al. (2002) [16], ^b^ Selby et al. (2008) [29], ^c^ Giesen-Bloo (2010) [11]. CFI = Comparative Fit Index; TLI = Tucker-Lewis Index; RMSEA = Root Mean Square Error of Approximation; SRMR = Standardized Root Mean Square Residual; AIC = Akaike Information Criterion; BIC = Bayesian Information Criterion. Lower χ^2^, RMSEA, SRMR, AIC, and BIC values indicate better fit. The bifactor model demonstrated the best overall fit among the tested models, with acceptable error metrics (RMSEA < 0.08, SRMR < 0.09) despite modest CFI/TLI values.

**Table 4:** Group comparisons based on Mann–Whitney U tests of BPDSI total scores and their subscales.

Subscale	Patients_Mean (SD)	Controls_Mean (SD)	W Statistic	*p*
Abandonment	3.36 (2.46)	1.13 (1.15)	3323	<0.001
Relationships	3.01 (1.84)	1.14 (1.13)	3391	<0.001
Identity	1.59 (0.91)	0.9 (0.78)	2972	<0.001
Impulsivity	2.1 (1.71)	0.63 (0.58)	3383	<0.001
Para (suicide)	1.55 (1.69)	0.31 (0.59)	3333	<0.001
Affective Instability	7.29 (2.13)	4.64 (2.13)	3379	<0.001
Emptiness	6.91 (1.98)	4.14 (1.92)	3514	<0.001
Anger-control	2.71 (2.13)	1.14 (1.2)	3018	<0.001
Dissociation	3.22 (2.26)	1.44 (1.16)	3178	<0.001
BPDSI Total Score	31.76 (12.09)	15.48 (6.15)	3660	<0.001

**Note:** BPDSI-IV = Borderline Personality Disorder Severity Index—Fourth Version; SD = Standard Deviation; W = Mann–Whitney U test statistic. Subscales reflect DSM-5 BPD criteria assessed by the BPDSI-IV.

**Table 5:** Pearson correlations and partial correlations of the BPDSI-IV total score with other variables and test statistics.

	Total Group		BPD-Patients	
	r with BPDSI-IV Scores	Corrected r ^a^	r with BPDSI-IV Scores	Corrected r ^a^
BPD Checklist	0.7 ***	0.8 ***	0.62 ***	0.6 ***
SCL 90 R	0.7 **	0.74 ***	0.62 ***	0.6 ***
Rosenberg Scale	0.5 ***	0.5 ***	−0.7 ***	−0.5 ***
BSI-53	0.6 ***	0.58 ***	0.54 ***	0.5 **
DSQ-40 Mature Defenses	−0.45 **	−0.45 *	−0.16 **	−0.04
DSQ-40 Neurotic Defenses	0.42 **	0.4 **	0.14 **	0.1 **
DSQ-40 Immature Defecenses	0.5 ***	0.5 **	0.2 ***	0.2 ***
QOL-N1	−0.3 *	−0.3	−0.06 *	−0.06
QOL-N2	−0.4 **	−0.4 **	−0.2 *	−0.08 *
QOL-N3	−0.3 ***	−0.3 ***	−0.04 *	−0.01 *
QOL-N4	−0.28 ***	−0.28 ***	−0.22 *	−0.13 *

**Note:** * *p* < 0.05; ** *p* < 0.01; *** *p* < 0.001 (2-tailed). ^a^ Corrected for age, gender, education years. BPD Checklist: Borderline Personality Disorder Checklist; SCL 90 R: Symptom Checklist–90–R; BSI: Brief Symptom Inventory 53; DSQ-40: Defense Style Questionnaire-40; QOL-N1: Quality of Life from WHO-QOL, Physical health; QOL-N2: Quality of Life from WHO-QOL, Psychological health; QOL-N3: Quality of Life from WHO-QOL, Environment; QOL-N4: Quality of Life from WHO-QOL, Social relationships.

## Data Availability

The datasets presented in this article are not readily available because the data are part of an ongoing study. Requests to access the datasets should be directed to the corresponding author.

## Supplementary Materials

The following supporting information can be downloaded at https://www.mdpi.com/article/10.3390/jcm14113699/s1, Figure S1: Participant Flow Diagram, Table S1: STROBE Checklist for Cross-Sectional Studies.

## Abbreviations

The following abbreviations are used in this manuscript:

BPD	Borderline Personality Disorder
NSSI	Non-suicidal Self injury
BPDSI	Borderline Personality Disorder Severity Index
SCL-90R	Symptom Checklist 90 Revised
DSQ	Defense Style Questionnaire
BSI	Brief Symptom Inventory
SCID-5 PD	Structured Clinical Interview for DSM-V Personality Disorders
ICC	Intraclass Correlation Coefficients
RMSEA	Root Mean Square Error of Approximation
SRMR	Standardized Root Mean Square Residual
