# Supplementary material for: Psychometric Properties of the Greek Version of the BPDSI-IV: Insights into Borderline Personality Disorder Severity
Source: J Clin Med. 2025 May 25;14(11):3699. doi: 10.3390/jcm14113699 (PMC12156871; doi:10.3390/jcm14113699)
Supplement: Supplementary file 1 [file jcm-14-03699-s001.zip › Participant_Flow_Diagram_BPDSI-IV.pdf]

Supplementary Figure S1: Participant Flow Diagram

Participants assessed for eligibility at Personality Disorders Unit (n ≈ 200)

↓

Excluded (e.g., not meeting inclusion criteria or declined to participate) (n ≈ 40)

↓

Diagnosed with BPD and included in the study (n = 128)

↓

Healthy controls recruited from community (n = 32)

↓

Final sample included in psychometric validation (n = 160)
