# Supplementary material for: Psychometric Properties of the Greek Version of the BPDSI-IV: Insights into Borderline Personality Disorder Severity
Source: J Clin Med. 2025 May 25;14(11):3699. doi: 10.3390/jcm14113699 (PMC12156871; doi:10.3390/jcm14113699)
Supplement: Supplementary file 1 [file jcm-14-03699-s001.zip › STROBE_Checklist_BPDsi.pdf]

Table S1 ： STROBE Checklist for Cross-Sectional Studies

This checklist was completed for the manuscript titled "Validation of the Greek Version of the BPDSI-IV: Insights into Borderline Personality Disorder Severity" to ensure comprehensive and transparent reporting in accordance with the STROBE guidelines for observational studies.

Section / Topic	Item No.	Recommendation	Reported on Page(s)
Title and Abstract	1	Indicate the study’s design with a commonly used term in the title or the abstract.	Title, Abstract
		Provide in the abstract an informative and balanced summary of what was done and what was found.	Abstract
Introduction	2	Explain the scientific background and rationale for the investigation being reported.	pp. 1–2
	3	State specific objectives, including any prespecified hypotheses.	pp. 2–3
Methods	4	Present key elements of study design early in the paper.	pp. 2–3
	5	Describe the setting, locations, and relevant dates, including periods of recruitment and data collection.	pp. 2–3
	6a	Give the eligibility criteria, and the sources and methods of selection of participants.	pp. 2–3

	7	Clearly define all outcomes, exposures, predictors, potential confounders, and effect modifiers.	pp. 2–4
	8	For each variable of interest, give sources of data and details of methods of assessment (measurement).	pp. 2–4
	9	Describe any efforts to address potential sources of bias.	p. 3
	10	Explain how the study size was arrived at.	p. 2
	11	Explain how quantitative variables were handled in the analyses.	pp. 3–4
	12a	Describe all statistical methods, including those used to control for confounding.	pp. 3–4
	12b	Describe any methods used to examine subgroups and interactions.	pp. 3–4
	12c	Explain how missing data were addressed.	Not applicable
	12d	If applicable, describe analytical methods taking account of sampling strategy.	Not applicable
	12e	Describe any sensitivity analyses.	p. 4
Results	13a	Report numbers of individuals at each stage of study.	p. 3
	13b	Give reasons for non-participation at each stage.	p. 3 (if applicable)

	13c	Consider use of a flow diagram.	Not included
	14a	Give characteristics of study participants.	Table 1
	14b	Indicate number of participants with missing data for each variable of interest.	Not applicable
	15	Report numbers of outcome events or summary measures.	Tables 2, 3
	16a	Give unadjusted and, if applicable, confounder-adjusted estimates and their precision.	Table 3, p. 4
	16b	Report category boundaries when continuous variables were categorized.	p. 3 (age groups)
	16c	If relevant, consider translating estimates of relative risk into absolute risk.	Not applicable
Discussion	18	Summarize key results with reference to study objectives.	p. 4
	19	Discuss limitations of the study.	p. 5
	20	Give a cautious overall interpretation of results.	p. 5
	21	Discuss the generalisability (external validity) of the study results.	p. 5
Other Information	22	Give the source of funding and the role of the funders.	p. 6
